# Perfluorinated compounds linked to central precocious puberty in girls during COVID-19: an untargeted metabolomics study

**DOI:** 10.3389/fendo.2024.1491411

**Published:** 2024-12-24

**Authors:** Haidan Li, Manfang Xie, Hailing Luo, Yuhua Cai, Li Liu, Hongai Li, Yuanping Hai, Yi Ren, Jing Xue, Xiaojie He, Xiaoyan Huang, Wei Xiang

**Affiliations:** ^1^ Department of Pediatrics, Hainan Medical University School of Pediatrics, Hainan Women and Children’s Medical Center, Haikou, Hainan, China; ^2^ Department of Pediatrics, Hainan Women and Children’s Medical Center, Haikou, Hainan, China; ^3^ Department of Endocrinology and Metabolism, Shunde Hospital, Southern Medical University (The First People’s Hospital of Shunde), Foshan, Guangdong, China; ^4^ Department of Pediatrics, Hainan Modern Women and Children’s Hospital, Haikou, Hainan, China; ^5^ Department of Clinical Laboratory, Hainan Women and Children’s Medical Center, Haikou, Hainan, China; ^6^ Pediatrics Research Institute, The Second Xiangya Hospital of Central South University, Changsha, Hunan, China

**Keywords:** central precocious puberty, metabolomics, endocrine disruptors, perfluorinated compounds, children

## Abstract

**Background and objective:**

The incidence of central precocious puberty (CPP) in girls increased significantly during the COVID-19 pandemic. This study aimed to explore the impact of perfluorinated endocrine disruptors on CPP through metabolomics analysis in girls from Hainan Province, China.

**Methods:**

Serum samples from 100 girls with CPP and 100 healthy controls were collected. Untargeted metabolomics profiling was performed using ultra-high performance liquid chromatography coupled with quadrupole-Exactive Orbitrap mass spectrometry (UHPLC-Q-Exactive-Orbitrap-MS). Differentially expressed metabolites (DEMs) were screened, and pathway enrichment analysis was conducted.

**Results:**

Principal component analysis (PCA) and partial least squares discriminant analysis (PLS-DA) revealed distinct metabolic profiles between the CPP and control groups. A total of 511 metabolites were identified, including 296 up-regulated DEMs and 255 down-regulated DEMs. Three perfluorinated compounds—PFSM-perfluoroalkyl_sulfonamide_Me, PFSM-FSAA, and PFCA-unsaturated—were significantly upregulated in the CPP group. KEGG pathway enrichment analysis suggested the involvement of multiple pathways in the CPP process regulated by these compounds.

**Conclusions:**

Perfluorinated compounds may promote CPP in girls by interfering with various pathways and affecting the hypothalamic-pituitary-gonadal axis function. This study highlighted the need for further research and public health measures to address environmental endocrine disruptors.

## 1 Introduction

Central Precocious Puberty (CPP) is a common and complex endocrine disorder affecting an increasing number of children worldwide (1). Recent years have witnessed a significant surge in CPP incidence, particularly during the COVID-19 pandemic (2). This phenomenon has gathered widespread attention in the medical community, yet the precise underlying causes remain elusive (3). CPP not only leads to premature development of secondary sexual characteristics but also potentially exerts long-term adverse effects on children’s height development, psychological wellbeing, and social adaptability (1). Given the limited understanding of CPP etiology, an in-depth investigation of its pathogenesis and exploration of potential environmental factors, such as specific dietary compounds, are crucial. These efforts hold significant clinical importance for developing effective prevention strategies and improving patient outcomes.

Metabolomics is a discipline that studies the types, structures, quantities, changes, and functions of all metabolites within an organism. It can provide information about the metabolic state, activity, and regulatory mechanisms of metabolic pathways. Metabolomics can be used to investigate disease mechanisms, diagnose and predict disease risk, and assess the effects of drug treatments. Previous studies have utilized metabolomics approaches to investigate pubertal development in children. For instance, Fang et al. have found that pathways such as fatty acid synthesis, and gonadotropin-releasing hormone (GnRH) are closely associated with pubertal development based on an animal and clinical study (4). Wu et al. utilized a cross-platform metabolomics approach using nuclear magnetic resonance (NMR) and demonstrated that endocrine disruption caused by perfluorinated compound (PFC) exposure directly or indirectly drives metabolic changes and forms a global metabolic network disturbance in CPP (5). Therefore, metabolomics is a well-established method for studying precocious puberty in children. Finding reliable biomarkers through this approach has potential value.

This study aims to investigate the metabolomics of central precocious puberty in girls in Hainan Province, China, during the COVID-19 pandemic. Based on untargeted metabolomics, this study screened for differentially expressed metabolites (DEMs). Focusing on perfluorinated endocrine disruptors, the study analyzed the associated metabolites and performed pathway enrichment analysis to provide new insights on central precocious puberty in girls.

## 2 Materials and methods

### 2.1 Subjects

This case-control study included 100 serum samples from girls with CPP who were hospitalized and diagnosed in the Department of Genetic Metabolism and Endocrinology at Hainan Women and Children’s Medical Center from January 2020 to October 2022. These samples were used as the case group (CC group). During the same period, 100 serum samples were collected from healthy participants as the control group (NN group); this group exhibited normal development and were free from any diseases. There were no statistically significant differences in age and BMI between the two groups (**Table 1**). This study was approved by the Medical Ethics Committee of Hainan Women and Children’s Medical Center [Approval No. (2020-(010)]. Samples and clinical information were collected after obtaining informed consent from the patients’ guardians.

**Table 1:** Feature of included cases.

Features	NN (n=100)	CC(n=100)	P-value
Age (Year)	8.15 ± 0.50	8.20 ± 0.68	0.49
Height (cm)	127.85 ± 4.97	133.62 ± 7.02	<0.001
Weight (Kg)	28.44 ± 4.96	31.44 ± 6.59	<0.001
BMI (kg/m^2^)	17.33 ± 2.35	17.48 ± 2.64	0.66
Basic LH(IU/L)	NA	4.28 ± 4.15	NA
LH^Peak^/FSH^Peak^ (Ratio)	NA	1.46 ± 0.93	NA
Bone Age (Year)	NA	9.91 ± 1.17	NA

### 2.2 Inclusion and exclusion criteria

#### 2.2.1 Inclusion criteria

Case group inclusion criteria: age < 11 years, female, diagnostic criteria for central precocious puberty (1).

#### 2.2.2 Exclusion criteria

The following subjects were excluded: those with significant clinical symptoms, severe underlying diseases, recent infections (within six months), recent antibiotic use (within three months), chronic gastrointestinal issues or surgeries, glucose metabolism disorders, or autoimmune connective tissue diseases.

### 2.3 Sample collection and clinical data collection

Samples from both the case group and the control group were collected by nurses from the Department of Genetic Metabolism and Endocrinology at Hainan Women and Children’s Medical Center. Blood was collected in centrifuge tubes and allowed to clot and separate by standing at 37°C (or room temperature) for 1 hour. The samples were then centrifuged at 3000 g for 10 minutes at room temperature. The supernatant (1 ml) was aliquoted into 1.5 mL centrifuge tubes and stored at -80°C. Once all the samples were collected, they were used for serum metabolomics testing. Basic information and examination data were also collected from the control and case groups; they include gender, age, height, weight, BMI, bone age, basal LH value, and the LH peak/FSH peak ratio from the GnRHa stimulation test, The dietary habits of the study participants were analyzed and summarized in **Table 2**.

**Table 2:** Baseline characteristics of the dietary survey.

Variables	CC(n=100)	NN(n=100)	χ2/t	P value
Puffed snacks intake (%)			57.2	<0.001
None	2	16		
Occasional	45	78		
Often	46	5		
Every day	7	1		
fried foods intake (%)			23.3	<0.001
None	8	20		
Occasional	67	77		
Often	22	3		
Every day	3	0		
canned foods intake (%)			15.6	0.001
None	5	8		
Occasional	49	70		
Often	40	22		
Every day	6	0		

### 2.4 Sample preparation and quality control

The protocol outlined the preparation of samples for metabolic profiling involving LC-MS analysis. A 100 µL sample was mixed with 400 µL methanol/acetonitrile (1:1 v/v) via vortexing, then sonicated in ice baths for 1 hour to ensure thorough extraction. Afterward, it was incubated at -20°C for 1 hour and centrifuged at 4°C for 20 minutes at 14,000 g to separate and collect supernatants. These were then dried under vacuum. Quality control (QC) samples, created by pooling aliquots from all samples, were processed similarly for data normalization. Dried extracts were reconstituted in 50% acetonitrile, filtered through a 0.22 µm filter, and stored in 2 mL HPLC vials at -80°C until analysis.

### 2.5 LC–MS/MS analysis and data processing

The UHPLC-MS/MS analysis for metabolomics profiling was conducted using a UPLC-ESI-Q-Orbitrap-MS system involving a Shimadzu Nexera X2 LC-30AD coupled with a Q-Exactive Plus. Separation was achieved with an ACQUITY UPLC^®^ HSS T3 column (2.1×100 mm, 1.8μm) at a flow rate of 0.3 mL/min, using 0.1% formic acid in water and 100% acetonitrile as mobile phases. The gradient profile lasted 15.1 minutes, including linear increases in buffer B to 100% and a re-equilibration period. HESI source conditions included a spray voltage of 3.8kv (positive) and 3.2kv (negative), among others. Full MS scans were acquired at 70,000 resolution, MS/MS scans at 17,500, with specified injection times and collision energies. QC samples were injected every six samples. Data was processed using MS-DIAL, with specific criteria for metabolite identification and feature selection, ensuring data quality and reliability.

### 2.6 Multivariate statistical analysis and KEGG enrichment analysis

The multivariate statistical analysis for metabolomics data was conducted using R version 4.0.3 and relevant R packages. The data underwent Pareto scaling for mean-centered normalization. Key statistical approaches included principal component analysis (PCA), orthogonal partial least-square discriminant analysis (OPLS-DA), and partial least-square discriminant analysis (PLS-DA). These models were evaluated for overfitting using permutation tests and they were assessed based on R2X (cumulative), R2Y (cumulative), and Q2 (cumulative) values, with perfect models having values close to 1. OPLS-DA helped identify discriminating metabolites via the variable importance on projection (VIP) score, with values greater than 1 deemed significant.

Significant metabolites were determined with VIP scores over 1.0 and a p-value less than 0.05 from a two-tailed Student’s t-test. ANOVA was used for multiple group analyses, and fold changes were calculated between classes. Identified metabolites underwent cluster analysis using R.

For pathway analysis, KEGG enrichment analysis was performed on differential metabolites to identify disrupted biological pathways, using the KEGG database. A Fisher’s exact test was employed for evaluating pathway enrichment, and results were adjusted for multiple testing using FDR correction, with pathways considered significant if p < 0.05.

### 2.7 Pathway analysis of endocrine-disrupting compounds

By analyzing the correlation between metabolites and endocrine-disrupting compounds (PFSM-perfluoroalkyl_sulfonamide_Me, PFSM-FSAA, and PFCA-unsaturated) in each sample, with a correlation coefficient of 0.5 as the threshold, metabolites that were correlated with endocrine-disrupting compounds were obtained (**Supplementary File S1**). To visualize the results, a Sankey diagram was employed to effectively illustrate the complex relationships and flow patterns among the identified metabolic pathways. The Sankey diagram was generated using the pyecharts library in the R programming language.

### 2.8 Data analysis

In this study, data was shown in Mean +/- SD. Data screening and processing were performed using SPSS 23.0 software, including fold change analysis, chi-square test, and t-tests. An OPLS-DA model was used to obtain variable importance for projection (VIP) values. Metabolites with VIP > 1 were considered preliminary differential metabolites between groups. T-tests further verified the significance, selecting metabolites with VIP > 1 and p-value < 0.05 as significant differential metabolites.

## 3 Results

### 3.1 Principal component analysis

**Figure 1:**
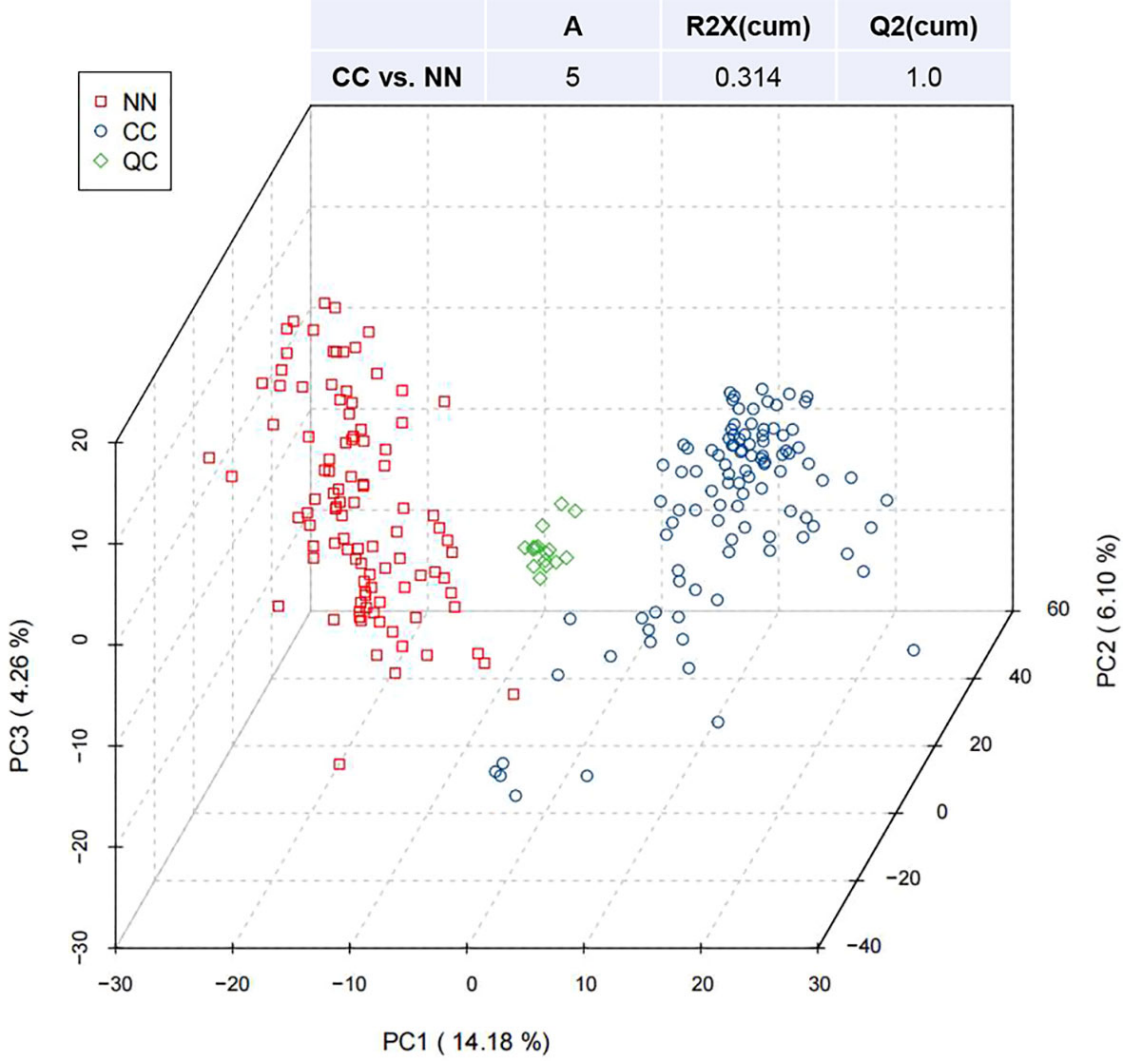
Partial least squares discriminant analysis (PLS-DA) of metabolites in the CC and NN groups.

**Figure 1** presented the PCA of all samples, including QCs. The scatter plots of samples from both the CC and NN groups were within the 95% confidence interval, indicating overall stable quality control. Additionally, the CC and NN group samples were maximally separated, suggesting that the analytical equipment used was stable and the experimental data were reliable.

### 3.2 Partial least squares discriminant analysis

**Figure 2:**
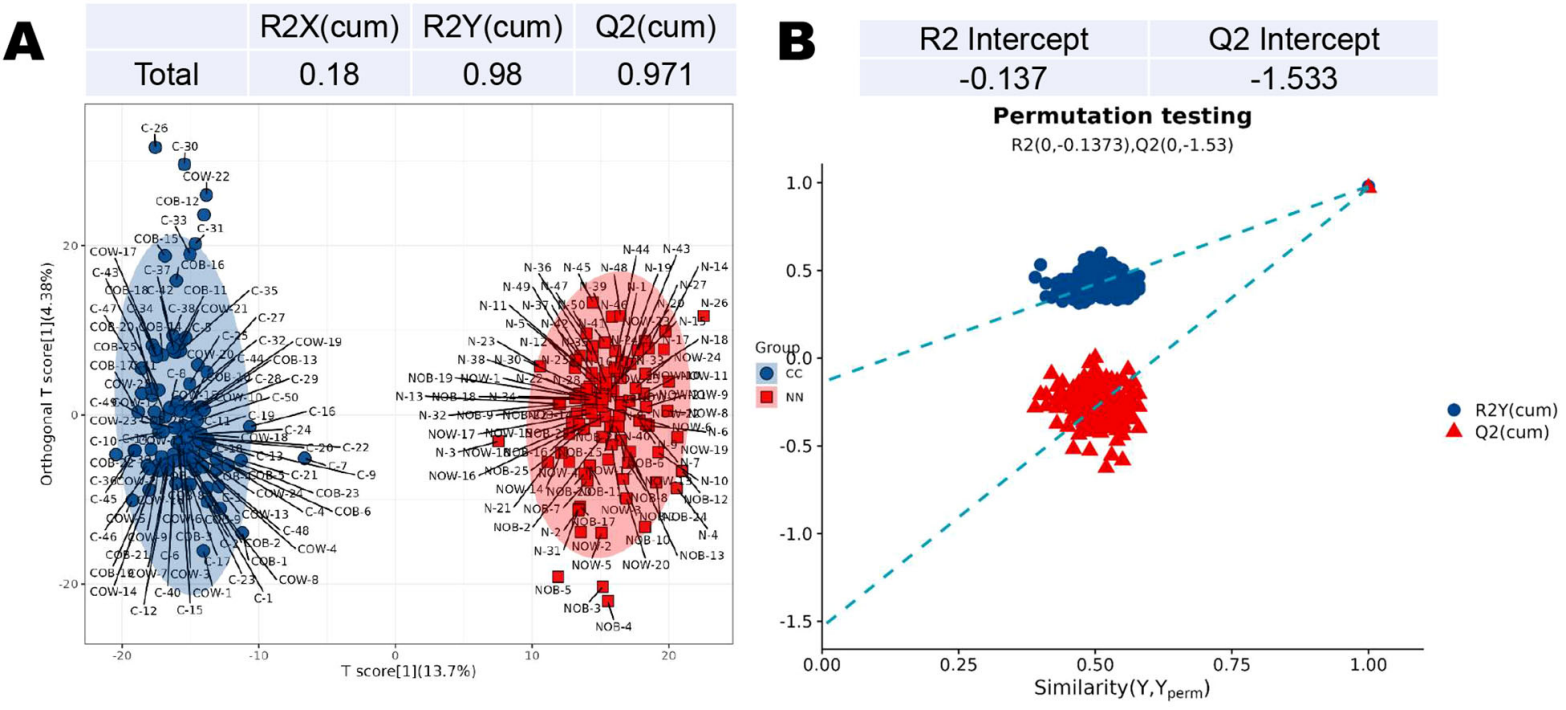
Partial least squares discriminant analysis (PLS-DA) of metabolites in the CC and NN groups. **(A)** The intercepts of R2 and Q2 were 0.98 and 0.97, respectively. **(B)** Permutation analysis revealed a Q2 value less than 0 (Q2=-1.533), indicating no overfitting in the model.

In the PLS-DA, the intercepts of R2 and Q2 are 0.98 and 0.97, respectively (**Figure 2A**). The permutation analysis revealed a Q2 value less than 0 (Q2=-1.533), indicating no overfitting in the model, thus confirming its reliability and effectiveness (**Figure 2B**).

### 3.3 Differential metabolites and pathway analysis

**Figure 3:**
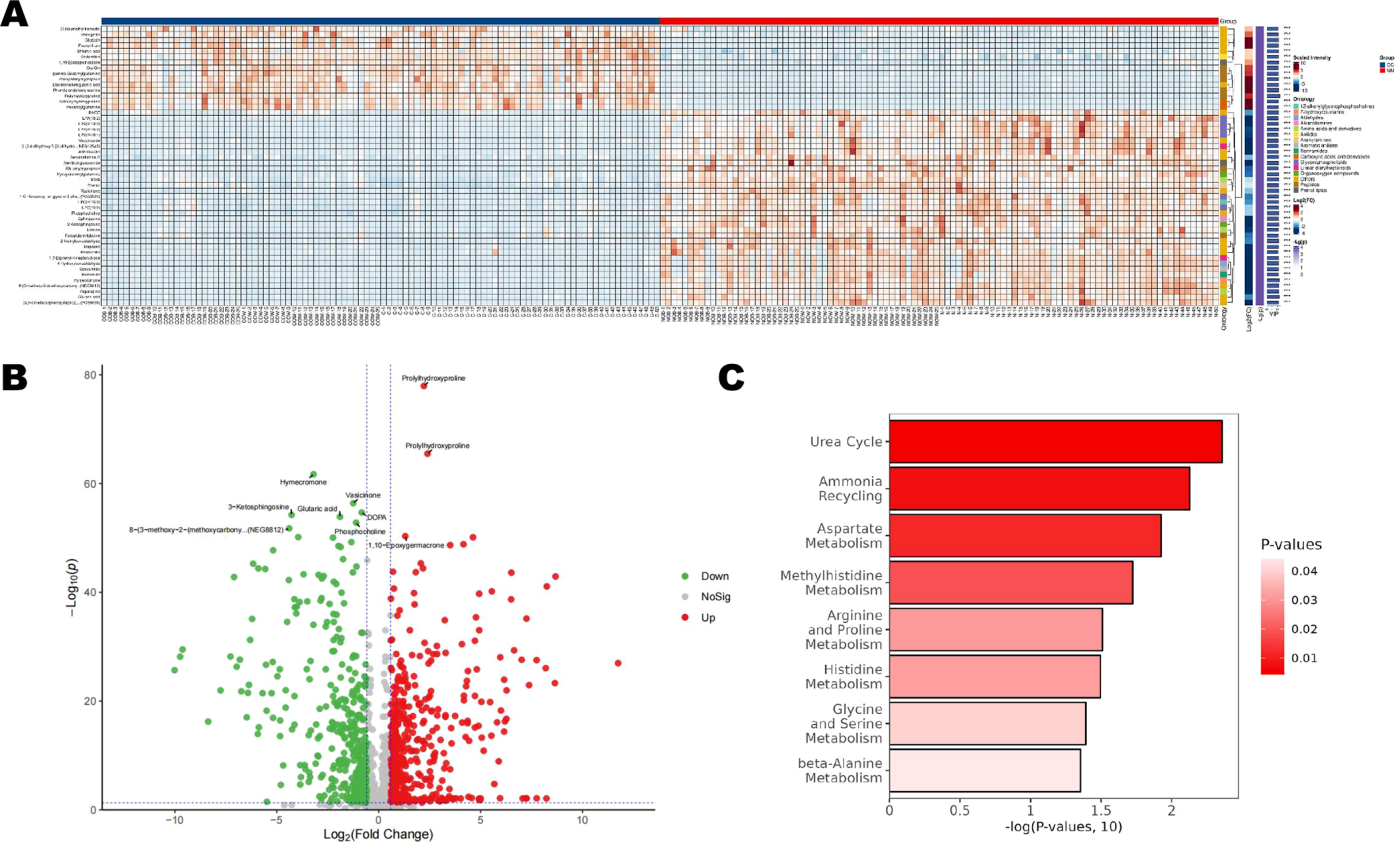
Differential metabolites and pathway analysis between the CC and NN groups. **(A)** A total of 551 metabolites were identified **(B)**, including 296 upregulated metabolites, and 255 downregulated metabolites were in the CC group. **(C)** SMPDB pathway bubble chart displaying the significantly enriched pathways (p<0.05).

A total of 551 metabolites were screened (**Supplementary File S2**; **Figure 3A**). Among them, 296 metabolites—including Prolyihydroxyproline, Inosine, and Epoxygermacrone—were upregulated in the CC group, while 255 metabolites—such as Hymecromene, Vasicinone, and Glutaric acid—were downregulated in the CC group (**Figure 3B**). **Figure 3C** displays the significantly enriched (p<0.05) SMPDB pathway bubble chart. The x-axis represented the percentage of matched differential metabolites while the y-axis indicated the negative logarithm transformation of the P-value. The bubble fill color ranged from dark red to light, representing increasing P-values and decreasing significance. The results suggested potential associations between the differential metabolites and pathways such as the Urea cycle, Ammonia recycling, Aspartate Metabolism, and Methylhistidine Metabolism (**Figure 3C**).

### 3.4 Endocrine-disrupting compounds analysis

**Figure 4:**
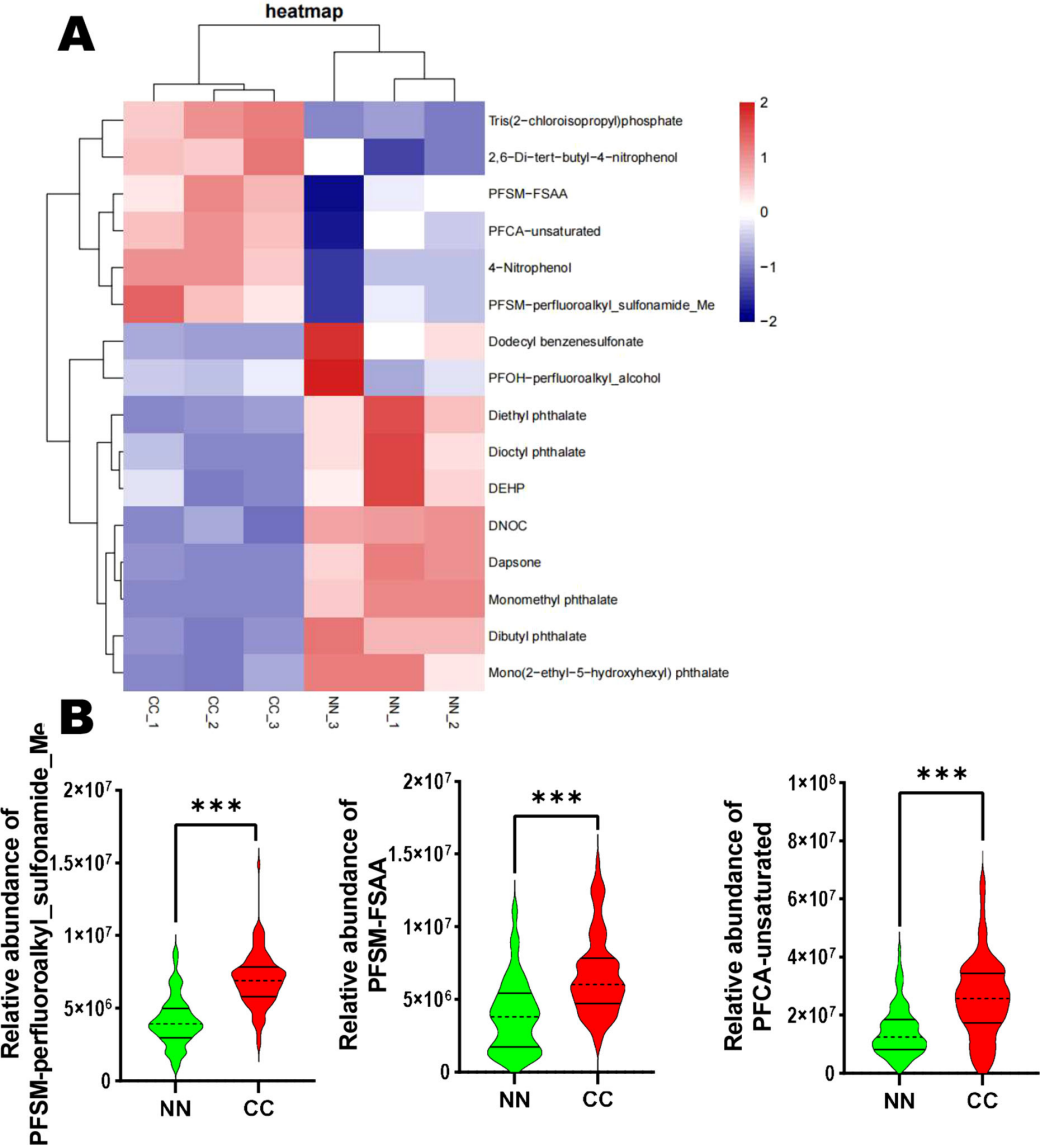
Endocrine-disrupting compounds analysis in the CC and NN groups. **(A)** Among the 10 disruptors, 7 compounds were downregulated, while 3 compounds (PFSM-perfluoroalkyl_sulfonamide_Me, PFSM-FSAA, and PFCA-unsaturated) were significantly upregulated in the CC group. **(B)** Relative abundance analysis of perfluorinated compounds. ***P<0.001 vs. NN group.

Perfluorinated compounds and phthalates have been shown to disrupt normal endocrine function. In this study, we focused on 10 disruptors, including Monomethyl phthalate. The analysis revealed that 7 compounds—namely Monomethyl phthalate, Dibutyl phthalate, Mono(2-ethyl-5-hydroxyhexyl) phthalate, Diethyl phthalate, Dioctyl phthalate, PFOH-perfluoroalkyl_alcohol, and DEHP—were downregulated in the CC group. In contrast, 3 compounds—including PFSM-perfluoroalkyl_sulfonamide_Me, PFSM-FSAA, and PFCA-unsaturated—were significantly upregulated in the CC group (**Figure 4A**). Furthermore, we performed a relative abundance analysis of perfluorinated compounds (PFOH-perfluoroalkyl_alcohol, PFSM-perfluoroalkyl_sulfonamide_Me, PFSM-FSAA, and PFCA-unsaturated), and the results are shown in **Figure 4B**. Specifically, PFSM-perfluoroalkyl_sulfonamide_Me, PFSM-FSAA, and PFCA-unsaturated were significantly upregulated in the CC group, while PFOH-perfluoroalkyl_alcohol was significantly downregulated in the CC group (**Figure 4B**).

### 3.5 Pathway enrichment analysis of endocrine-disrupting compounds

**Figure 5:**
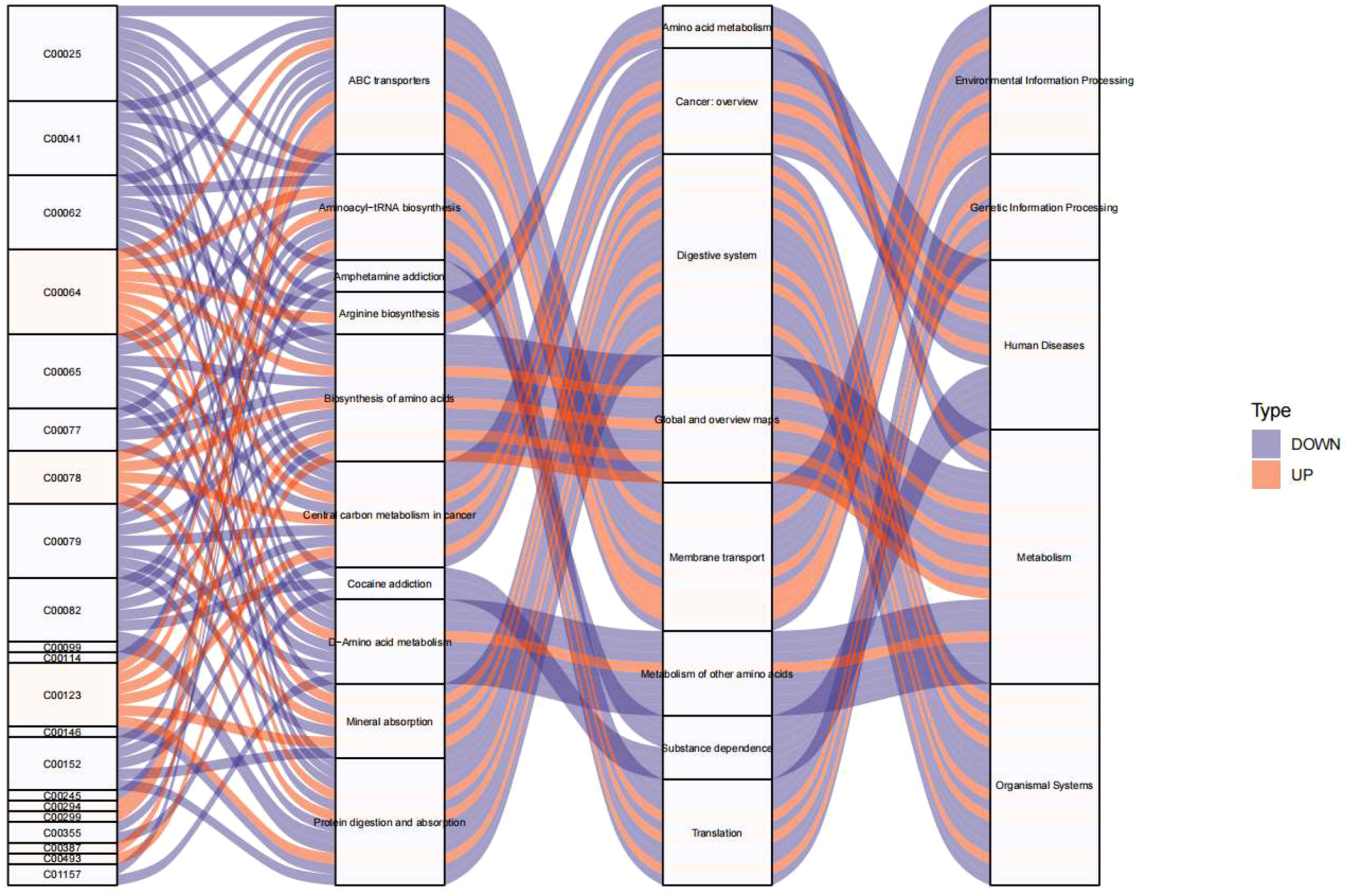
KEGG pathway enrichment analysis visualized as Sankey diagram of endocrine-disrupting compounds (PFSM-perfluoroalkyl_sulfonamide_Me, PFSM-FSAA, and PFCA-unsaturated) associated metabolites.

To elucidate the metabolic pathways involved in the presence of endocrine-disrupting chemicals (PFSM-perfluoroalkyl_sulfonamide_Me, PFSM-FSAA, and PFCA-unsaturated), KEGG analysis was performed based on the metabolites associated with these three endocrine disruptors. As shown in **Figure 5**, Environmental Information Processing (including ABC transporters and Neuroactive ligand-receptor interaction), Genetic Information Processing (including Aminoacyl-tRNA biosynthesis), Human Diseases (including Central carbon metabolism in cancer), Metabolism (including D-Amino acid metabolism, Phenylalanine metabolism, Arginine biosynthesis, Alanine, aspartate and glutamate metabolism, Phenylalanine, tyrosine and tryptophan biosynthesis, Glycine, serine and threonine metabolism, Taurine and hypotaurine metabolism, and 2-Oxocarboxylic acid metabolism), and Organismal System (including Protein digestion and absorption and Mineral absorption) may be involved in the central precocious puberty process regulated by the three endocrine-disrupting chemicals, PFSM-perfluoroalkyl_sulfonamide_Me, PFSM-FSAA, and PFCA-unsaturated.

## 4 Discussion

The COVID-19 pandemic and associated lockdown measures have led to significant changes in dietary patterns and lifestyles, which are closely related to the increased incidence of CPP (6). As an illustrative example, during the pandemic, residents’ food preferences shifted, with a significant increase in the consumption of processed and canned foods (7).These foods often contain preservatives, plasticizers, and other food additives, some of which have been proven to have endocrine-disrupting effects. These additives can promote the occurrence of CPP by activating estrogen receptors and interfering with the synthesis and metabolism of sex hormones (8). Moreover, the pandemic has led to prolonged sedentary time and reduced physical activity in children, which may exacerbate childhood obesity (9). The present study employed an untargeted metabolomics approach to analyze the association between precocious puberty in girls and endocrine-disrupting chemicals in Hainan Province. The aim was to reveal potential risks for the development of precocious puberty in girls and provide new insights and strategies for prevention and intervention insofar as this condition is concerned.

Perfluorinated compounds (PFCs) are a class of synthetic chemicals containing multiple carbon-fluorine bonds; they are widely used in industrial production and daily life. However, PFCs have characteristics such as resistance to degradation, high mobility, and strong bioaccumulation. Consequently, they tend to accumulate through the food chain and ultimately enter the human body. PFSM and PFCA are a class of PFCs that can bind to estrogen receptors and mimic the effects of estrogen. Additionally, these compounds can interfere with the synthesis, transport, and metabolism of thyroid hormones, causing thyroid dysfunction (10). There is evidence to show that PFC levels in the human body are associated with factors such as drinking contaminated water, contact with food and food packaging materials, and indoor dust. Once they enter the human body, PFCs can disrupt endocrine system functions through various mechanisms (11). In addition to these mechanisms, studies have found that PFCs can also activate peroxisome proliferator-activated receptors (PPARs), affecting lipid metabolism and energy balance (12). Furthermore, numerous studies have shown that PFC exposure is associated with various health issues, such as reproductive developmental abnormalities, metabolic disorders, and neurobehavioral disorders. Wu et al. found a direct association between precocious puberty in girls and PFC exposure. Their study, based on clinical CPP patients, analyzed the serum levels of PFCs in this population and found that estradiol and prolactin were significantly associated with PFCs in CPP patients. In terms of clinical phenotype, PFCs exhibited clear characteristics of driving CPP and inducing metabolic disorders (5). Our study found associations between specific perfluorinated compounds (PFCs)—namely PFSM-perfluoroalkyl_sulfonamide_Me, PFSM-FSAA, and PFCA-unsaturated—and CPP in children. While these findings are suggestive, further research is needed to confirm any causal relationship. Future studies should focus on more extensive environmental monitoring and population exposure assessments of PFCs to better understand their potential impact on children’s health.

Through our dietary investigation, we found that the precocious puberty group had significantly higher intake of canned foods, fried foods, and packaged puffed snacks compared to the control group. These food categories often involve packaging materials that may release perfluorinated compounds when subjected to high temperatures during processing, storage, or consumption, potentially increasing exposure risks (13). Combining our research findings that the precocious puberty group had significantly higher levels of perfluorochemicals than the control group, it suggests a close association between perfluoroalkyl sulfonamide Me (PFSM), PFSM-FSAA, PFCA-unsaturated, and central precocious puberty in children. Research has indicated that the exposure to chemicals such as phthalates and bisphenol A, widely used in plastic products, may be associated with health issues such as precocious puberty (14).Therefore, it is necessary to strengthen environmental monitoring and population exposure assessment of PFCs and take effective measures to reduce the adverse effects of PFCs on children’s health. Future investigations should focus on three critical aspects: (1) comprehensive monitoring of perfluorinated compounds in the identified packaging materials, as indicated by **Table 2**; (2) rigorous validation of the putative role of these compounds in accelerating pubertal onset; and (3) elucidation of the molecular mechanisms underlying their potential effects on precocious puberty. These targeted research directions will be instrumental in advancing our understanding of the complex relationship between perfluorinated compound exposure and central precocious puberty in children.

Multiple pathways affected by PFSM-perfluoroalkyl_sulfonamide_Me, PFSM-FSAA, and PFCA-unsaturated may be involved in the process of precocious puberty in girls. Metabolomic analysis in the present study identified three perfluorinated compounds (PFSM-FSAA, PFCA-unsaturated, and PFSM-perfluoroalkyl_sulfonamide_Me) that may potentially influence the function of the hypothalamic-pituitary-gonadal (HPG) axis and promote the occurrence of precocious puberty in girls through various pathways. These pathways include Environmental Information Processing, Genetic Information Processing, Human Diseases, Metabolism, and Organismal System. For instance, ABC transporters have the capacity to influence the hormone balance of the HPG axis by regulating the utilization of cellular steroid hormones, ultimately exerting a decisive impact on estrogen production and affecting the process of sexual maturation (15). Studies have shown that neuroactive ligands, such as gonadotropin-releasing hormone (GnRH), play a key role in the HPG axis by stimulating the pituitary gland to release gonadotropins, which in turn regulate gonadal function and hormone production (16). Zhang et al. demonstrated that the Neuroactive ligand-receptor interaction pathway was enriched in children with central precocious puberty, which is similar to the findings of the present study (17). More importantly, the three perfluorinated compounds were enriched in metabolic pathways closely related to the HPGA or central precocious puberty, including D-Amino acid metabolism, Phenylalanine metabolism, Arginine biosynthesis, and Alanine, aspartate, and glutamate metabolism. These pathways participate in the synthesis and metabolism of various neurotransmitters and neuromodulators, thereby regulating the excitability of GnRH neurons, influencing the synthesis and release of GnRH, and ultimately modulating HPG axis function and contributing to the process of precocious puberty (18). In addition to these mechanisms, these pathways can also indirectly affect HPG axis function by influencing energy metabolism and growth and development. For example, the 2-Oxocarboxylic acid metabolism pathway involves the synthesis and metabolism of key metabolic intermediates such as oxaloacetate and α-ketoglutarate, participating in the regulation of multiple central metabolic pathways, including glycolysis, the tricarboxylic acid cycle, and amino acid metabolism (19), thereby involving in the regulation of precocious puberty (20). In the present study, by analyzing the pathways associated with endocrine-disrupting chemicals, we aimed to provide insights into the mechanisms of central precocious puberty. However, this study did not perform qualitative or quantitative detection of metabolites and pathways, which requires further research and in-depth exploration.

In summary, using an untargeted metabolomics approach, this study found that three PFCs were associated with CPP in girls, suggesting that these PFCs may promote the occurrence of CPP by interfering with pathways such as environmental information processing and affecting HPG axis function. This study revealed the potential molecular mechanisms of PFCs leading to CPP, providing new insights for prevention and intervention in the case of CPP in girls. Nonetheless, the metabolomics results still need to be validated by other omics data. Additionally, the specific pathways through which PFCs affect the HPG axis require further elucidation.

## Data Availability

The datasets presented in this study can be found in online repositories. The names of the repository/repositories and accession number(s) can be found in the article/**Supplementary Material**.
